# Does it matter in what family constellations adolescents live? Reconsidering the relationship between family structure and delinquent behaviour

**DOI:** 10.1371/journal.pone.0265964

**Published:** 2022-04-13

**Authors:** Robert Svensson, Björn Johnson

**Affiliations:** 1 Department of Criminology, Malmö University, Malmö, Sweden; 2 Department of Social Work, Malmö University, Malmö, Sweden; University of South Florida, UNITED STATES

## Abstract

**Objectives:**

This study examines the associations between ten family structure types and delinquency, including four groups of symmetrical and asymmetrical living arrangements. We also adjust for attachment to parents and parental monitoring.

**Methods:**

Data are drawn from four cross-sectional surveys conducted between 2016 and 2019 in southern Sweden. The sample consists of 3,838 adolescents, aged 14–15. Negative binomial models were used to calculate the associations between family structure and delinquency.

**Results:**

The results show that those living in single-father, single-mother, father-stepmother, mother-stepfather families report significantly more delinquency than adolescents living with both their parents. Adolescents living in “symmetrical” family arrangements, i.e. both parents are single or have a new partner, reported lower levels of delinquency, whereas adolescents living in “asymmetrical” family arrangements, i.e. where either the mother or the father, but not both, have a new partner, reported higher levels of delinquency. Most of the associations between family structure and delinquency decline when adjusted for attachment to parents and parental monitoring.

**Discussion:**

This study shows that it is important to move on to the use of more detailed categorisations of family structure in relation to delinquency. We need to increase our knowledge about the group of adolescents that moves between parents and especially about the different constellations of asymmetrical and symmetrical living arrangements.

## Introduction

Family structure is commonly considered to be a factor that should be taken into consideration in analyses of delinquency. A number of studies using cross-sectional and longitudinal designs have shown that not living with both parents is positively associated with delinquency [1–5]. Not living with both parents has also been found to be associated with other negative outcomes, for example mental health problems, lower levels of well-being and high levels of alcohol use [6–9].

Although most scholars have used a rather simplistic measure of family structure, i.e. living with both parents vs. not living with both parents, some have used a more detailed categorisation of family structure in relation to delinquency. For example, research has shown that adolescents living in single parent families (with mother or father) and with stepparents report higher levels of delinquency compared to adolescents living with both their mother and father [2, 4, 10–12]. One limitation of these previous studies is that they do not consider the relatively large and increasing [13, 14] group of adolescents who move between parents, i.e. have alternating living arrangements. In this study, we examine how such alternating living arrangements are associated with delinquency among adolescents, an issue that has not previously been examined in criminological research.

These family constellations are those in which adolescents have alternating living arrangements, adolescents move between parents, thus spending most of their time residing equally (or fairly) with both parents, and both parents often have equal access rights (often referred to as joint physical custody) [13–15]. This means that they are moving between two households that may be, but are not necessarily, in different geographical areas, and that the parents may also have different rules [16]. Previous research shows that children who move between their parents generally report higher levels of well-being and have a better relationship with their mother and father than those who mainly live with a single parent [9, 17–19]. Relatively little research has to date examined the situation of different sub-groups among those with alternating living arrangements, e.g., if the mother lives alone while the father lives with a new partner, or if both biological parents now live in homes that also include a stepparent. Fallesen & Gähler found that adolescents from “symmetrical” alternating living arrangements, i.e. where both parents are single or (ii) where both are living with a new partner, reported similar levels of well-being to adolescents living in intact families [20]. However, they also found that the level of well-being appears to be lower among adolescents with “asymmetrical” alternating living arrangements, i.e. (iii) where the mother is single and the father is living with a new partner, or (iv) where the father is single and the mother is living with a new partner, and that this seems to be more pronounced if the mother is single and the father is with a new partner.

How can we understand the association between family structure and delinquency? Although several explanatory models have been discussed [1, 2, 21, 22] it has generally been argued that family processes may be seen as the key mediator between not living with both parents and negative outcomes [8, 23, 24]. Family processes are often defined as emotional bonds with parents, parental monitoring or discipline [24, 25]. In line with social control theory, both the emotional bond between child and parents (indirect control) and parental monitoring (direct control) are associated with delinquency [26–29]. The less strongly attached a child is to its parents, the less the child may care about their expectations and he or she thus becomes “free to deviate” [26], and the less controlled a child is by its parents the higher is the likelihood of delinquency [30]. In this context, it has been argued that family structure is important, but mainly indirectly, and that the association between family structure and delinquency mainly work through the social control exercised via attachment to parents and parental monitoring [1, 11].

Empirically, some studies have examined the association between family structure, family process and delinquency among adolescents. For example, previous research shows four family structure groups to be significantly associated with delinquency, but after adjusting for a number of family process variables (such as parental control and emotional bonds), none of the family structure variables were significant [11]. In addition, all of the family process variables were associated with delinquency. In line with this, it was found that the association between single parent households and delinquency becomes weaker after adjusting for family process variables [29]. On the other hand, Vanassche et al. did not find any empirical evidence that the association between family structure (stepparents and/or living with a single parent) and delinquency decreased after adjusting for family process measures [4]. Similarly, previous research found weak empirical evidence that family factors mediate the association between a broken home and delinquency [31].

To sum up, it is well-known that not living with both parents is significantly associated with delinquency [2]. Although some studies have examined how different family structure types are associated with delinquency, the results are somewhat mixed, and no previous study has used a more detailed categorization of groups and focused specifically on examining the importance of alternating living arrangements. Finally, a number of studies have examined the association between family structure types and delinquency, adjusting for family process variables. This research indicates that family structure variables is indirectly associated with delinquency, but once again the empirical findings are mixed and none of the existing studies have examined these associations using a more detailed categorization of family structure.

Against this background, the present study has two overall aims, (i) to examine the association between ten family structure types and delinquency, and (ii) to examine the association between the different categories of family structure and delinquency, adjusting for two central family process variables—parental monitoring and attachment to parents. As far as we know, no previous study has focused on delinquency using such a detailed categorization of family structure and especially including four groups of symmetrical and asymmetrical living arrangements.

## Material and methods

### Participants

The study is based on secondary data analysis from the Öckerö project [32, 33], an evaluation of an alcohol prevention program [34]. The project included an annual, anonymously completed, cross-sectional online self-report survey, which was conducted at 17 secondary schools in eight small municipalities in the county of Skåne. Skåne is Sweden’s most southerly county, with a population of approximately 1.4 million. The eight municipalities have between 13,000 and 19,000 inhabitants, a total of 125,000 altogether. The survey was conducted in all classes in years 7–9 (i.e. 12–15 years of age), the final three years of compulsory education. The survey was conducted at the beginning of the autumn term in each of four successive years, 2016–2019.

In this study, we employ data on adolescents in year nine (aged 14–15) from the years 2016 to 2019. We decided to use data only for those aged 15 (year 9), since very few of the younger adolescents reported delinquency. The surveys included a total of 1,018 adolescents in 2016, 1,028 in 2017, 1,050 in 2018 and 1,018 in 2019. The four subsamples combined produce a total sample of 4,114 adolescents. This represents a response rate of 86.7%. After listwise deletion of missing values on the variables of interest, our final sample size below is based on 3,838 cases i.e., a loss of 6.7% (n = 276). The missing data is fairly evenly distributed across the included variables. We considered employing an imputation procedure, but since most of the variables included are based on single items, we decided against this.

The research design and study procedures were approved by the Regional Ethical Review Board at Lund University.

### Measures

#### Dependent variable

*Self-reported delinquency* measures whether the respondents has committed any of the following nine offences during the past 12 months: (1) hit someone; (2) robbed someone; (3) carried a knife as weapon when going out; (4) shoplifting; (5) stolen a bicycle; (6) stolen something from someone’s pocket or bag; (7) stolen something from a car or broken into a car to steal something; (8) graffiti; (9) damage. Response alternatives is for each crime type: never = 0; 1 time or more = 1. The nine items were combined into a *variety scale* that counts the number of nine different behaviours involving acts of delinquent behaviour. The variety scale is seen as the most appropriate measure of delinquency and in general it has higher reliability and validity than frequency measures [35, 36]. The measure is heavily skewed with 68% of the respondents reporting no offence.

#### Independent variables

*Family structure* is a measure based on a combination of the two questions: (1) Do you live with your mother and your father? (response alternatives: yes / only father / only mother / sometime mother, sometime father / either with mother or father) (2) Do you live with any stepparent? (response alternatives: no / yes, with my stepfather / yes, with my stepmother / yes, with both stepfather and stepmother). Of the different categories within these two questions, we created a measure of family structure including the following ten groups: (1) living with mother and father; (2) living with father; (3) living with mother; (4) living with father and stepmother; (5) living with mother and stepfather; (6) alternating living: father and mother, i.e. symmetrical living arrangements; (7) alternating living: mother and father, stepmother, i.e. asymmetrical living arrangements; (8) alternating living: father and mother, stepfather, i.e. asymmetrical living arrangements; (9) alternating living: father and mother, stepmother and stepfather (i.e. both stepparents), i.e. symmetrical living arrangements. To create the four groups of alternating living we used the following answer alternative:”sometime mother, sometime father” alone or in combination with”with stepfather” and”stepmother” in different combinations. (10) other living constellation. The ten variables will be used as dummy variables in the regression models, with living with mother and father specified as the reference category.

*Attachment to parents* is an additive index based on five items (1) Do you feel your mother/father trusts you? (2) Do you usually feel that your mother/father gives you support and encouragement? (3) Do you think you have a good contact with your mother/father? (4) Do you feel that your mother/father cares about you? (5) Can you usually talk about everything (for example problems) with your mother/father? (response alternatives: no, never / not much / rarely / sometimes / often / yes, always). The alpha for the scale is .90. This scale measures the strength of the emotional relationship between child and parents and a high score indicates that respondents have strong emotional bonds to their parents.

*Parental monitoring* is an additive index comprised of five items: (1) Do you have specific times when you have to be home in the evenings? (2) Do you have to ask your parents for permission to go out in the evening? (3) Do you have to contact your parents if you are not home by a certain time? (4) If you are going out in the evening, do you have to tell your parents who you are going to meet? (5) If you are going out in the evening, do you have to tell your parents what you are going to do? (response alternatives: no, never / rarely / sometimes / often / yes, always). Alpha for the scale is .81. This index measures whether parents are actively doing anything to keep track of their children’s whereabouts, and a high score indicates high levels of parental monitoring.

Four *control variables* are employed. *Gender* is coded as zero for girls and one for boys. *Born in Sweden* is coded as 0 if the respondent is born abroad and 1 if the respondent is born in Sweden. *Older sibling* is coded as zero for not having an older sibling and one for having an older sibling. *Year of study* represents the year when the study was conducted and is used in the form of four dummy variables. Year 2016 is the reference category employed in the analyses.

For a description of the measures employed see Table 1. The table shows that the majority live with both their mother and father (66.9%). A smaller proportion of the respondents live with a single father than a single mother (1.7% vs. 7.4%). A larger proportion of the respondents live with a mother and a stepfather than with a father and a stepmother. 15.4% live in an alternating living arrangement, i.e. moving between parents. Of the four alternating living arrangements, the most common is that in which the respondents live with a single mother and a single father (6.6%).

**Table 1:** Descriptive statistics (N = 3,838).

	Mean/%	SD	Min	Max
Delinquency variety	.76	1.58	0	9
Family structure				
Mother & father	66.9%	-	0	1
Single father	1.7%	-	0	1
Single mother	7.4%	-	0	1
Father & stepmother	1.2%	-	0	1
Mother & stepfather	4.8%	-	0	1
AL: mother & father ^a^	6.6%	-	0	1
AL: mother & father, stepmother ^b^	2.4%	-	0	1
AL: father & mother, stepfather ^b^	3.5%	-	0	1
AL: father & mother, both stepparents ^a^	2.9%	-	0	1
Other living constellation	2.5%	-	0	1
Attachment to parents *	21.65	3.80	5	25
Parental monitoring *	18.65	4.81	5	25
Gender (1 = boys)	50.0%	-	0	1
Born in Sweden (1 = yes)	83.4%	-	0	1
Older siblings (1 = yes)	63.2%	-	0	1
Year of study				
2016	24.5%	-	0	1
2017	25.0%	-	0	1
2018	26.0%	-	0	1
2019	24.6%	-	0	1

Note: AL = alternating living.

* standardized in regression models

^a^ Symmetrical living arrangements

^b^ Asymmetrical living arrangements

### Statistical analysis

First, we compared differences between the family structure groups with regard to delinquency, attachment to parents and parental monitoring using one-way analysis of variance. Second, we specify two negative binomial models using the delinquency variety scale as the outcome. We use negative binomial modelling because our outcome is a skewed count variable with overdispersion [37, 38]. In the first model we include the different family structure types as dummy variables, with living with mother and father as the reference category. In this model we also add our three social background variables, i.e. gender, older sibling and born in Sweden and the year in which the study was conducted (with year 2016 as reference category). In the second model we add the two family process variables, i.e. attachment to parents and parental monitoring to the model.

In order to examine whether the association between the nine family structure types and delinquency declines significantly when adjusting for attachment to parents and parental monitoring, we estimate a cross-model test between model 1 and model 2 [39, 40]. The model combines the parameter estimates and covariance matrices of the two models into a single parameter vector and simultaneous covariance matrix, on which the cross-model test can be estimated. The cross-model comparison is based on a Chi-square test of cross-model coefficient differences. All tests examined are based on df = 1, i.e., we test one variable at a time. The *suest* command in the Stata software package was used [41, 42].

As the data are based on respondents who are clustered in schools, clustered robust standard errors are presented for the negative binomial models [43, 44]. Since the number of clusters are rather few a sensitivity test was performed using cluster bootstrap standard errors and no changes in the main results was found [44, 45]. Interval-scaled predictors have been standardized prior to inclusion in the regression models. All statistical analyses have been conducted in Stata/SE version 17.

## Results

Initially we compare the differences between the family structure groups in relation to the delinquency measure and the independent variables using analysis of variance tests. The results show clear differences between the ten groups in relation to delinquency (see Table 2). First, adolescents living with both mother and father have been involved in delinquency to a lesser extent than the other groups. The results also show that delinquency is more common among those adolescents living in single-parent households and with stepparents. Adolescents living with both parents have in general stronger bonds to their parents, whereas adolescents in alternating living arrangements seem to have similar bonds to their parents as those who live with both parents.

**Table 2:** Means/proportions of variables by family structure (N = 3,838).

	Both parents n = 2568	Single father n = 67	Single mother n = 284	Father & stepmoth. n = 45	Mother & stepfather n = 183	AL: mother & father^a^ n = 255	AL: mother & father, stepmoth.^b^ n = 92	AL: father & mother, stepfath.^b^ n = 135	AL: father & mother, both steppar.^a^ n = 112	Other living const. n = 97	F-test / Chi-square
Delinquency variety	.63	1.22	1.02	1.07	1.14	.71	1.14	1.06	.84	1.77	10.80***
Attachment to parents	22.38	19.19	19.12	18.81	18.59	21.96	21.61	21.14	21.71	18.34	59.69***
Parental monitoring	18.91	16.49	18.35	19.00	18.73	18.04	17.95	17.49	18.32	17.92	4.38***
Gender (1 = boys) (%)	50.5	56.7	44.4	51.1	38.3	54.5	45.7	47.4	51.8	63.9	25.95**
Born in Sweden (%)	82.8	82.1	77.1	88.9	78.7	94.9	95.7	96.3	97.3	50.5	155.02***
Older siblings (%)	61.4	76.1	75.0	66.7	57.4	67.1	57.6	60.7	66.1	73.2	36.05***

Note: AL = alternating living. Low values on the family measures indicate weak bonds to parents, being weakly controlled by parents.

^a^ Symmetrical living arrangements

^b^ Asymmetrical living arrangements

** p < .01,

*** p < .001.

Results from the negative binomial regression analyses are presented in Table 3. In the first model, the results show that living with single father, single mother, with father and stepmother and with mother and stepfather are all positively associated with delinquency. Among the four alternate living arrangements, the pattern is clear. Living alternately where both parents are single or where both are living with a new partner is not associated with delinquency. However, the other two alternatives, i.e. living alternately where one of the parents is single while the other parent is living with a new partner, are both positively associated with delinquency.

**Table 3:** Relationship between family structure and delinquent behaviour (variety scale). Negative binomial regression.

	Model 1			Model 2			^a^ Cross-model comparison
	Coef.	*p*	IRR	Coef.	*p*	IRR	
Family structure (ref: mother and father)							
Single father	.641	.012	1.898	.143	.484	1.154	20.93***
Single mother	.508	< .001	1.661	.203	.068	1.225	30.01***
Father & stepmother	.474	.032	1.606	.019	.943	1.019	5.91*
Mother & stepfather	.715	< .001	2.044	.357	.016	1.429	20.93***
AL: mother & father ^c^	.103	.198	1.108	-.001	.995	.999	1.60
AL: mother & father, stepmother ^d^	.715	< .001	2.045	.609	< .001	1.839	1.40
AL: father & mother, stepfather ^d^	.542	.004	1.719	.344	.085	1.410	3.66
AL: father & mother, both stepparents ^c^	.295	.130	1.343	.208	.278	1.231	3.43
Other living constellation	.957	< .001	2.604	.466	.036	1.593	8.13**
Attachment to parents				-.435	< .001	.647	
Parental monitoring				-.387	< .001	.679	
Controls ^b^	Yes			Yes			
Log pseudolikelihood	-4289.401			-4112.0491			
N	3,838			3,838			

Note: AL = alternating living. Coef. = Unstandardized regression coefficient. IRR = Incidence rate ratio. The p-values are calculated using robust standard errors, clustered by schools.

^a^ This column presents whether the coefficients for the family structure types are significantly different between Model 2 and Model 1 using chi-square (df = 1) test (i.e., cross-model coefficient differences).

^b^ Control variables included in the model are gender, born in Sweden, older siblings, year of study.

^c^ Symmetrical living arrangements

^d^ Asymmetrical living arrangements

* p < .05,

** p < .01,

*** p < .001.

In the second model, attachment to parents and parental monitoring are both negatively associated with delinquency. The results also show that most of the family structure variables become weaker and insignificant when adjusting for the family process variables. For example, the coefficient for living with a single father declines significantly from .641 to .143 and that for living with a single mother decline significantly from .508 to .203. Although the coefficient for living with a mother and a stepfather declines significantly from .715 to .357, it is worth mentioning that this association remains significant when adjusting for attachment to parents and parental monitoring.

The association between delinquency and living alternately with one’s mother and one’s father and stepmother remains significant (p = < .001), and the coefficient does not change significantly between Model 2 and Model 1. The association between delinquency and living alternately with one’s father and one’s mother and stepfather is no longer significant, and the coefficient does not change significantly between Model 2 and Model 1. Finally, the associations for a total of five of the nine variables decline significantly when adjusting for the family process variables. Interestingly, the four that did not change were all categories of alternating living arrangements.

As a robustness check we repeated our models using logistic regression focusing on estimates of average marginal effects for delinquency (no act vs. one or more acts) and violence (no act vs. one or more acts). The results followed a pattern similar to that obtained using frequency of delinquency.

## Discussion

This study has examined the associations between ten categories of family structure and delinquency, adjusting for attachment to parents and parental monitoring. As far as we know, no previous study has examined the association between so many different family structure arrangements and adolescent delinquent behaviour, particularly with regard to the inclusion of four groups of asymmetrical and symmetrical living conditions.

First, we found that adolescents living with only their father, only their mother, father and stepmother, mother and stepfather, and in the category other living constellations report higher levels of delinquency compared to adolescents living together with both their parents. In addition, we found some interesting differences between the groups. Living with single parents and living with a single mother and a stepfather were all positively associated with delinquency. These results are in line with previous research showing that living with single parents and stepparents is positively associated with delinquency [2, 11]. The results also show the importance of separating the analysis on the basis of whether youths live only with their mother or their father, a finding which has not been reported in most other studies [2, 4].

Second, we found that that the four alternating living arrangement to be differently associated with delinquency. Adolescents living in the two symmetrical arrangements, i.e. where both parents are single or both parents have new partners did not reported higher levels of delinquency than those living with both their parents, whereas adolescents living in the two asymmetrical arrangements, i.e., where one parent is single and the other has a new partner, reported higher levels of delinquency than those living with both their parents, particularly for those living with a mother and father and a stepmother. These findings are in line with previous studies focused on well-being, which show that results vary depending on the type of alternating living arrangement [20].

Third, we found that the strength of the association between the different family structure types (single father, single mother, stepmother, stepfather, other living constellations) and delinquency would become weaker when adjusting for attachment to parents and parental monitoring. The decline in the strength of the association between the family structure types and delinquency is in line with some previous research [11, 29], but contradicts the findings of other studies [4].

Finally, we found that the coefficient for the four alternating family arrangements did not decrease significantly when adjusting for attachment to parents and parental monitoring. Interestingly, the results show that the association between living alternatively with one’s mother and one’s father and stepmother is still significant when adjusting for attachment to parents and parental monitoring. This shows that the measures of attachment to parents and parental monitoring are not enough to explain the difference in the association between asymmetric living conditions and delinquency. In general, the findings are in line with the theoretical assumption that the association between the different family structure types declines when adjusting for attachment to parents and parental monitoring [8, 11, 23, 24]. However, this is not clear for the asymmetrical family living arrangement. One consequence for this could be that some important theoretical variables such as economic resources or measures of frustration and/or family conflict [2, 46, 47] are not included in the analysis. Overall, these findings indicates that we need to develop our understanding of these two groups in a theoretical point of view.

For youths who grow up in *asymmetric* alternating households, i.e. where one parent has a new partner but not the other, “may reflect situations more prone to animosity” and the family climate would not assume to be in the same balance found in symmetric households (Fallesen & Gähler, 2020:342). The imbalance that may be expected between children and parents might be due to one parent having met a new partner, and this in turn affecting the child. This may in turn lead to children allying themselves with the parent who has not met someone new. It may also be the case that the parent who is first to meet a new partner is ascribed responsibility for “difficulties” in the parents getting back together, which may create an imbalance in the family that would be likely to affect the family dynamic.

Further, the results from this study show the importance of separating and examining different family structure arrangements in more detail when examining the association with delinquency. However, one key question remains, and that is how we should understand the different findings noted in relation to asymmetric and symmetric alternating living arrangements. Could it be that the child has not accepted the new family member? Or could it be that the child thinks the relationship is unequal when the mother is single and the father has a new partner? These are questions that need to be examined further and where more research is thus needed. Another important question for further research would be to examine how children may be affected when a new child comes into the family arrangement, and how this affects behaviour in relation to various forms of delinquency. Another question of interest for future research is that of when the acts of delinquency occur: is it mainly when they are residing in one of their two households, or does it occur in connection with residency in both households?

A number of limitations need to be addressed. First, the data are based on a cross-sectional design, and we are therefore unable to draw conclusions about causality. It would therefore be important for future research to replicate these findings using longitudinal data. Second, there is also a need to include variables that would make it possible to examine other theoretical questions with regard to family economic resources (SES), stepsiblings and whether there are conflicts within the family [2, 20], and criminogenic indicators as for example delinquent friends, self-control and moral values [48–50]. Third, in this study the number of cases in some of the different family structure categories is rather small, and larger scale surveys are needed to explore the stability of the results. Using larger scale surveys, will also give the possibility to examine these associations for boys and girls and to examine whether the different family structure groups interact with attachment to parents and parental monitoring. Fourth, given our results focusing on delinquency, and the findings of other studies focusing on well-being in relation to different joint physical custody family constellations, there is a need to examine other outcomes such as use of alcohol, tobacco, and other drugs. Fifth, adolescents living in alternating family arrangements may integrate less among peers if the parents live far apart. This spatial dimension is also an interesting aspect to include in future studies. Sixth, an interesting topic for future research would be to distinguish between attachment to and monitoring by the mother and to the father and to examine whether it makes a difference with which parent the relationship is not so good.

Even given these limitations, however, this study shows that it is important to move on to the use of more detailed categorisations of family structure in relation to delinquency and to increase our knowledge about the group of adolescents that moves between parents and especially about the different constellations of asymmetrical and symmetrical living arrangements.
